# The clinical aspects of pituitary tumour genetics

**DOI:** 10.1007/s12020-021-02633-0

**Published:** 2021-02-04

**Authors:** Judit Dénes, Márta Korbonits

**Affiliations:** 1 Divison of Endocrinology, 2nd Department of Medicine, Health Center, Hungarian Defence Forces, Budapest, Hungary; 2 Centre for Endocrinology, William Harvey Research Institute, Barts and the London School of Medicine, Queen Mary University of London, London, UK

**Keywords:** Pituitary, Tumour, Genetics, FIPA, MEN

## Abstract

**Background:**

Pituitary tumours are usually benign and relatively common intracranial tumours, with under- and overexpression of pituitary hormones and local mass effects causing considerable morbidity and increased mortality. While most pituitary tumours are sporadic, around 5% of the cases arise in a familial setting, either isolated [familial isolated pituitary adenoma, related to AIP or X-linked acrogigantism], or in a syndromic disorder, such as multiple endocrine neoplasia type 1 or 4, Carney complex, McCune–Albright syndrome, phaeochromocytoma/paraganglioma with pituitary adenoma, DICER1 syndrome, Lynch syndrome, and USP8-related syndrome. Genetically determined pituitary tumours usually present at younger age and show aggressive behaviour, and are often resistant to different treatment modalities.

**Subject:**

In this practical summary, we take a practical approach: which genetic syndromes should be considered in case of different presentation, such as tumour type, family history, age of onset and additional clinical features of the patient.

**Conclusion:**

The identification of the causative mutation allows genetic and clinical screening of relatives at risk, resulting in earlier diagnosis, a better therapeutic response and ultimately to better long-term outcomes.

## Introduction

Consideration of genetic abnormalities in a patient with a pituitary tumour has only recently entered the clinical thinking of the practising endocrinologist. While in families with multiple endocrine neoplasia type 1 (MEN1) syndrome it was long recognised that members can develop pituitary adenomas with incomplete penetrance, the flurry of conditions we can now list with a genetic cause of pituitary adenoma would have been well beyond imagination 20 years ago. Indeed, most pituitary tumours are sporadic, but approximately 5% can be due to a hereditary disease. Pituitary tumours can occur in a familial setting, either isolated [Familial Isolated Pituitary Adenoma (FIPA), for example aryl hydrocarbon receptor-interacting protein (*AIP*) mutation-positive FIPA, or in patients with X-linked acrogigantism (XLAG)], or as part of a syndromic condition, such as MEN1 or MEN4, Carney complex (CNC), McCune–Albright syndrome (MAS), phaeochromocytoma/paraganglioma with pituitary adenoma (*3P* associations, 3Pa), DICER1 syndrome and a USP8-related syndrome and some other rare conditions where the nature of association with pituitary adenomas needs further studies (Table 1).

**Table 1:** Genetic alterations in pituitary tumours

	Abnormality	Germline	Mosaic mutation	Somatic (tumour only)
Isolated	*AIP*	*x*		
	*GPR101*	*x*	*x*	
	Unknown germline alterations gene(s) in *AIP*-negative FIPA	*x*		
Syndromic	*MEN1*, *CDKN1B*, *CDC73*^a^	*x*	*x*	
	*PRKAR1A*, *PRKACB*^a^	*x*		
	*SDHx*	*x*		*x*
	*MAX*	*x*		
	*DICER1*	*x*		
	*USP8*	*x*^a^		*x*
	*USP48*			*x*
	*MLH, PMS2*	*x*		
	*GNAS*		*x*	*x*
	*VHL*	*x*^a^		
	*RET*	*x*^a^		
	*TSC1*	*x*^a^		
Somatic	*SF3B1*			*x*
	*ATRX*			*x*

^a^Case reports/further study needed.

Genetic testing might lead to the recognition of a syndromic disease and therefore beneficial effects of timely identification of other aspects of the disease, or it can diagnose disease in family members at an early stage leading to earlier diagnosis and treatment, and ultimately to better outcomes.

Here we approach this issue from the practical point of view, centring the discussion on the presentation of the patient.

### Gigantism

The most common cause of a germline genetic abnormality in a patient with a pituitary adenoma is growth hormone (GH) excess, especially childhood-onset GH excess leading to gigantism. While the usual cause of acromegalic gigantism is a GH-secreting pituitary adenoma, resulting in elevated GH- and insulin-like growth factor 1 (IGF-1) levels, we should remember other conditions with tall stature, either as a normal variant or part of a syndrome with no abnormalities in the GH axis [1].

For true acromegalic gigantism we need to consider the age of onset of rapid growth. In case of very early onset, XLAG (<2 years of age) and MAS (from 3 years of age) might be the diagnosis.

XLAG is an infant-onset gigantism syndrome caused by germline or somatic mosaic duplication of the *GPR101* gene, which encodes a G-protein coupled receptor [2, 3]. The onset of accelerated growth is always observed before the age of 5 years, and in most patients during the first year of life. Apart from rapid growth, these children have acromegalic features, such as acral enlargement and facial coarsening and other abnormalities, like increased appetite and hyperinsulinemia [4]. Their pituitary MRI can show a tumour, a diffusely enlarged gland suggesting hyperplasia or a normal MRI. The tumours often have an unusual histological appearance (sinusoidal, lobular and acinar architecture, microcalcification and pseudo-follicles are characteristic), while hyperplasia can be seen on histology in some cases [3]. Pituitary hyperplasia can be also observed in same cases of MAS, CNC and in GH-releasing hormone (GHRH)-induced GH excess. *GPR101* variants, other than gene duplication, have not been associated with GH-secreting or other type of pituitary tumours [3, 5–8].

AIP-related gigantism is the most common genetic cause of pituitary gigantism [3, 9]. Patients typically show signs of the disease during the second decade but several cases have been described with accelerated growth already in the first decade. These patients usually have large invasive tumours needing multiple treatment modalities [10, 11], although microadenoma cured after surgery has also been described [12]. Pituitary apoplexy is a characteristic phenomenon in some *AIP* mutation-positive patients [11–13]. Male predominance is observed in *AIP* mutation-positive gigantism, although the physiologically later puberty and ascertainment bias due to taller stature in males could play a part in this.

MEN1 syndrome extremely rarely manifests as gigantism. A 5-year-old boy was described with a mammosomatotroph macroadenoma causing gigantism [14], while another case of MEN1-related gigantism was due to a pancreatic GHRH-secreting tumour [15].

MAS is a mosaic disease, caused by a mutation in the *GNAS* gene at an early post-zygotic stage of development. The variable phenotype depends on what tissues are affected by the mutation. MAS is characterised by fibrous dysplasia, cafè-au-lait spots of the skin and different endocrinopathies. Acromegaly affects around 20% of MAS patient. The mean age at diagnosis of acromegaly is 24.4 years; however, the disease can start in early childhood as well [16].

**Fig. 1:**
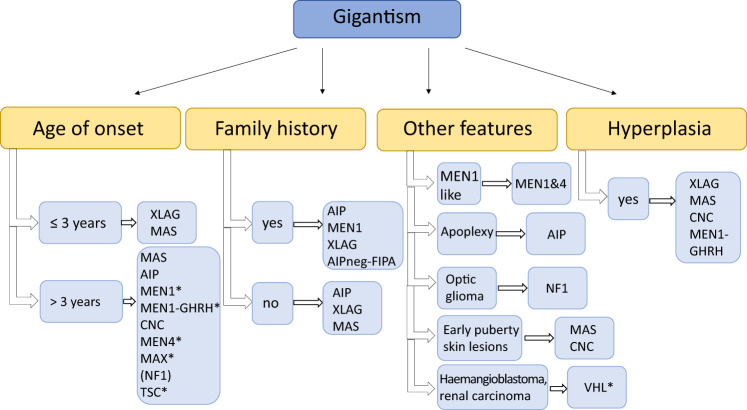
Diseases to be considered in cases of gigantism. AIP aryl hydrocarbon receptor-interacting protein, AIPneg-FIPA AIP mutation-negative Familial Isolated Pituitary Adenoma (this group does not represent XLAG families), CNC Carney complex, GHRH growth hormone-releasing hormone, MAS McCune–Albright syndrome, MEN multiple endocrine neoplasia, NF1 neurofibromatosis type 1, TSC tuberous sclerosis, VHL von Hippel–Lindau syndrome, XLAG X-linked acrogigantism. *Case reports/further study needed [69]

In the case of disease onset at >3 years of age but still in childhood, AIP, MEN1 and 4, CNC, MAS, 3Pa and a non-pituitary condition neurofibromatosis type 1 (NF1), should be considered. In NF1, approximately 10% of cases with optic glioma have GH excess often manifesting in childhood and causing accelerated growth; the exact mechanism of the GH excess is unknown (Fig. 1).

### Acromegaly

Apart from age of onset, the other major significant feature for GH excess patients is the family history. A positive family history is very suggestive of a genetic disease, but we should be aware of phenocopies—the same phenotype by chance and not due to common genetic background; this has been described in several *AIP*- and succinate dehydrogenase (*SDHx*)-positive families already [17–19]. In the case of a positive family history, the most common diseases are related to *AIP* or *MEN1* mutations. If there is no family history of pituitary tumours, this could be due to (i) a lack of genetic background, (ii) low penetrance, (iii) lack of information regarding a positive family history, (iv) a de novo germline or mosaic mutation and (v) imprinting complicating penetrance (*SDHD* for example).

FIPA is defined as two or more members of a family who develop pituitary adenoma with no other clinical manifestation. In FIPA families, either heterogeneous—with different pituitary tumour subtypes, or homogeneous—the most frequent pituitary adenoma subtypes are somatotrophinomas or prolactinomas. Germline mutations in the *AIP* gene have been identified in 10% of FIPA families, while in most cases the causative gene(s) remain unknown. It is important to note that *AIP* mutations can also occur in subjects with apparently sporadic early-onset somatotrophinomas or prolactinomas, as a consequence of incomplete penetrance, while de novo mutations are extremely rare. *AIP* mutation-positive tumours show a distinct phenotype, with younger age at diagnosis (usually age of onset under 30 years), tumour invasiveness and relative resistance to treatment with first-generation somatostatin receptor ligands. *AIP* mutation-positive patients usually need a multimodal therapeutic approach. In *AIP* mutation-negative patients somatotrophinoma is also the most common subtype (although not as common as in *AIP* mutation-positive subjects, around 78% compared to 58%) [10, 12, 18].

MEN1 is an autosomal dominantly inherited disease characterised by hyperparathyroidism, gastro-enteropancreatic neuroendocrine (NET) and pituitary tumours, with associated other endocrine and non-endocrine tumours. Mutations of the *MEN1* tumour suppressor gene are detected in 90% patients with the MEN1 phenotype. De novo germline or mosaic mutations occur in approximately 10% of cases. A pituitary tumour develops in 30–40% of the patients [20]. Prolactinomas are the most common clinically presenting pituitary tumour subtype, sometimes large and arising at a younger age. Patients with MEN1 may have plurihormonal adenomas. Non-functioning pituitary adenomas (NFPAs), somatotrophinomas and, rarely, corticotrophinomas or thyrotrophinomas have also been described. However, during systematic screening of *MEN1* mutation carriers, small non-functioning lesions, not dissimilar to incidentalomas, can often be found.

Around 10% of cases with a MEN1-like syndrome do not have a mutation in *MEN1*. Some of these patients harbour germline changes in the *CDKN1B* gene coding for the cyclin-dependent kinase inhibitor p27. This clinically overlapping rare condition is termed MEN4 [21, 22]. In a group of 24 patients with acromegaly and at least one other manifestation of MEN1 syndrome, but without pathogenic *MEN1* or *CDKN1B* variants, a *CDC73* missense mutation was found in one case [23].

In cases of the MEN1 syndrome, a GHRH-secreting pancreatic [24] or thymic [25] NET tumour might be part of the syndrome and the source of GHRH, which causes pituitary hyperplasia and thus acromegaly. A similar case has now been described in a patient with germline *MAX* mutation and multiple tumours including a GHRH-positive phaeochromocytoma [26].

CNC is a clinical diagnosis based on characteristic skin pigmentation, cardiac myxomas, primary pigmented nodular adrenocortical disease and pituitary tumour or hyperplasia, testicular lesions, melanotic schwannomas and others. Although around 75% of patients have an abnormal GH axis in CNC, only 10% of the patients show clinically acromegaly, and less frequently gigantism. Most patients (67%) have pituitary hyperplasia affecting the somatolactotroph cells, while only 10–12% of them have a real GH-producing pituitary tumour [27]. Genetic testing for inactivating germline mutations in the protein kinase A regulatory subunit 1-α (*PRKAR1A*) coding gene is available, and large deletions are known to cause more severe disease. There is a second locus located at 2p16, which is still unknown, but cytogenetic changes of the 2p16 chromosomal region are frequently observed in tumours from CNC patients [28, 29].

Phaeochromocytoma/paraganglioma can be associated with pituitary tumours (the 3Pa), even carcinoma [30], due to mutations in *SDHx* or *MAX* genes. The pituitary tumours include somatotrophinomas, prolactinomas and NFPAs. Recently *MAX* mutation has been described in families with phaeochromocytoma, pituitary adenoma and other endocrine- and non-endocrine tumours [26]. Acromegaly associated with phaeochromocytoma can be the consequence of a GHRH-secreting phaeochromocytoma without any pituitary adenoma [19, 31] (Table 2).

**Table 2:** Familial pituitary tumours or alterations, showing the syndrome, the background genetic alteration, the typical pituitary adenoma subtype and the other main clinical features in a syndromic setting

	Isolated	Syndromic							
Syndrome	FIPA	MEN1, 4	Carney complex	McCune–Albright	3Pa	Dicer syndrome	Neurofibromatosis	USP8-related syndrome	Lynch syndrome
Gene	*AIP*, *GPR101*	*MEN1*, CDKN1B, MAX*, CDC73*	PRKAR1A PRKACB*	*GNAS*	*SDHx*, *MAX*	*DICER1*	*NF1*	USP8*	MLH*, PMS2*
Type of pituitary tumour (secreted hormone)	GH GH&PRL PRL, NFPA, TSH*	PRL, NFPA, GH (ACTH, TSH)	GH, GH&PRLPRL, ACTH	GH, GH&PRL	PRL, GH, NFPA	ACTH	GH excess PRL*,^#^ ACTH*,^#^ NFPA*,^#^	ACTH	ACTH
Other main conditions	None	pHPT, pancreatic tumour, angiofibromas, adrenal adenomas, lipomas, meningiomas	Myxoma, PPNAD, pigmented lesions of the skin and mucosae	Fibrous dysplasia, hyper-functioning endocrinopathies, cafè-au-lait spots	Renal cell carcinoma, PGL/phaeo, GIST	PPB, goitre/thyroid cc, cystic nephroma, Sertoli–Leydig cell tumour	Neurofibromas, cafè-au-lait spots, optic glioma, phaeo	Developmental delay, dysmorphic features, lung and kidney disease	Colon, brain, uterine, pancreatic, ovarian, stomach tumour

*ACTH* adrenocorticotropic hormone, *GH* growth hormone, *GIST* gastrointestinal neuroendocrine tumour, *NFPA* non-functioning pituitary adenoma, *3Pa* 3P (phaeochromocytoma, paraganglioma and pituitary adenoma) association, phaeo, phaeochromocytoma, *pHPT* primary hyperparathyroidism, *PGL* paraganglioma, *PPB* pleuropulmonary blastoma, *PPNAD* primary pigmented nodular adrenocortical disease, *PRL* prolactin, *TSH* thyroid-stimulating hormone.*, case reports, ^#^, could be coincidences

NF1-associated GH excess should be considered in patients with characteristic clinical signs and symptoms of dermal neurofibromas, café-au-lait spots, axillary or inguinal freckling and hamartomas of the iris as well as brain neoplasms due to inactivating mutation of *NF1* gene. Around 10% of children with NF1 and optic pathways glioma show GH excess [32]. While tuberous sclerosis has been described with pituitary adenomas, the causative link between the two diseases is uncertain [33, 34].

Acromegalic features and GH hypersecretion have been recently described in patients with mutations in the X-linked immunoglobulin superfamily member 1 (*IGSF1*) gene. The exact mechanism of GH excess is unclear [35].

**Fig. 2:**
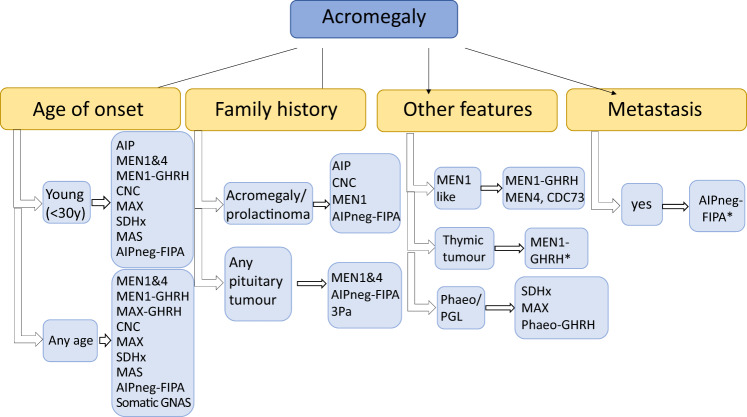
Diseases to be considered in cases of acromegaly. AIP aryl hydrocarbon receptor-interacting protein, AIPneg-FIPA AIP mutation-negative Familial Isolated Pituitary Adenoma (this group does not represent XLAG families), CNC Carney complex, FIPA Familial Isolated Pituitary Adenoma, GH growth hormone, GHRH growth hormone-releasing hormone, MEN multiple endocrine neoplasia, NET neuroendocrine tumour, phaeo phaeochromocytoma, SDH succinate dehydrogenase. *Case reports/further study needed [25, 70]

Somatic mutations can lead to acromegaly as well. The most frequent genetic alteration is somatic *GNAS* mutation, found in 40% of somatotrophinomas [36]. These tumours show favourable clinical features, such as older age at diagnosis, less invasiveness and better response to therapy (Fig. 2).

### Prolactinoma

**Fig. 3:**
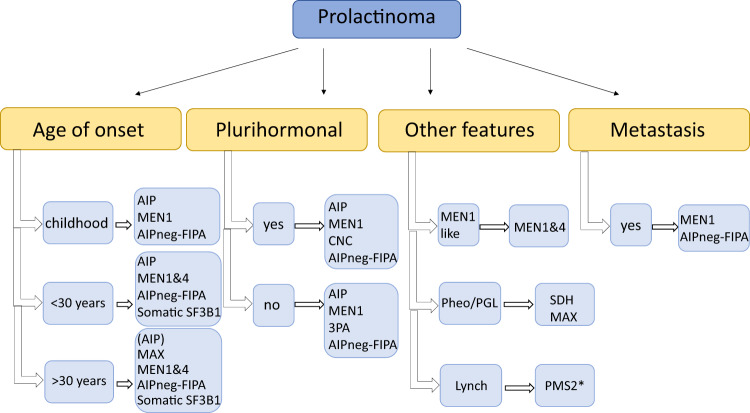
Diseases to be considered in cases of prolactinoma. AIP aryl hydrocarbon receptor-interacting protein, AIPneg-FIPA AIP mutation-negative Familial Isolated Pituitary Adenoma (this group does not represent XLAG families), MEN multiple endocrine neoplasia, NF1 neurofibromatosis type 1, 3Pa 3P (phaeochromocytoma, paraganglioma and pituitary adenoma) association, SDH succinate dehydrogenase. *Case reports/further study needed [45]

Prolactinomas are the most common pituitary adenoma subtype in MEN1, and the second most common in MEN4 and FIPA, both in *AIP*-positive (even as a homogenous prolactinoma family [37]) and *AIP*-negative FIPA, but can occur in *SDHx* cases as well (Fig. 3). Metastatic prolactinoma case has been described in MEN1 [38]. A large childhood-onset prolactinoma can be the first manifestation of *MEN1* or *AIP*-related disease. Recently, two families with *MAX* mutations with multiple tumour types were described, including a patient with prolactinoma and parathyroid tumour in one of them [26].

Recently, a somatic mutation in the *SF3B1* gene has been described in 20% of prolactinoma patients, showing higher prolactin (PRL) levels [39].

### Cushing’s disease

Infant-onset Cushing’s disease can occur in DICER1 syndrome with very low penetrance due to a pituitary blastoma, but this is pathognomic to the disease. Pituitary blastomas are aggressive tumours arising in young children, presenting clinically with often severe ACTH-dependent hypercortisolism. It is locally destructive, severe disease with high mortality [40]. A family history of young-onset large goitre, pleuropulmonary blastoma, Sertoli–Leydig cell tumours, nodular thyroid hyperplasia or differentiated cancer of the thyroid, cystic nephroma and renal sarcoma can be associated [41]. Cushing’s disease is rare (5–10%) in MEN1 [42], and to date no convincing *AIP* mutation-positive patient has been found with a corticotrophinoma. More recently, a few cases of *CDKN1B* mutations were described in children with corticotrophinoma with or without other features of MEN4 [43]. Germline potentially pathogenic *CABLES1* variants were identified in 2% of the cases in a patient cohort of Cushing’s disease [44]. Aggressively growing corticotroph tumours has been described as part of Lynch syndrome in patients with DNA mismatch repair genes *MSH6*, *PMS2* mutations and associated colorectal, endometrial, ovarian, urinary tract, small bowel, gastric, hepatobiliary, adrenocortical or malignant brain tumours [45]. Cushing’s disease has been described in patients with *RET* mutation as part of MEN2A or MEN2B syndrome. It is unclear whether these cases are coincidences, or *RET* mutation plays a causative role in pituitary tumour formation ([46, 47] and references within [19]).

**Fig. 4:**
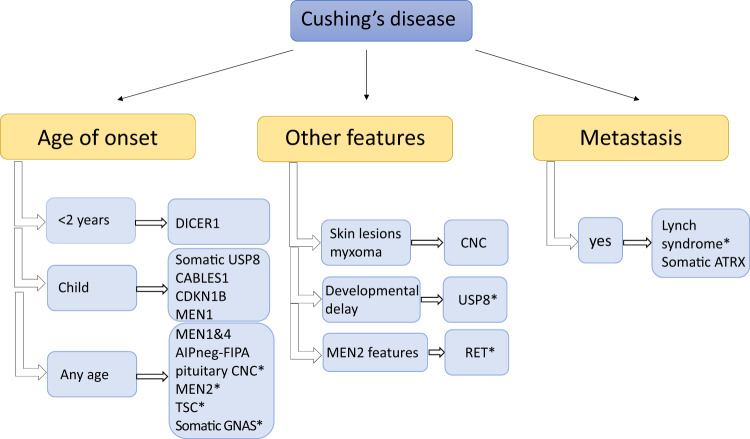
Diseases to be considered in cases of Cushing’s disease. AIP aryl hydrocarbon receptor-interacting protein, AIPneg-FIPA AIP mutation-negative Familial Isolated Pituitary Adenoma (this group does not represent XLAG families), CDKN1B cyclin-dependent kinase inhibitor 1B, CNC Carney complex, FIPA Familial Isolated Pituitary Adenoma, TSC tuberous sclerosis. *Case reports/further study needed [19, 45–47, 50, 52–54, 71–73]

Somatic *USP8* mutations can be observed in corticotrophinomas in a high (around 30%) percentage. Patients harbouring mutations in this gene are predominantly female, and they have more frequently microadenomas with better therapeutic outcome [48, 49]. Germline *USP8* mutation has been found in a paediatric case with recurrent Cushing’s disease and developmental delay, representing a syndromic form of pituitary adenomas [50]. Somatic *GNAS* mutation might occur very rarely in ACTH-producing pituitary adenomas [51, 52]. The differential diagnosis of Cushing’s syndrome is often challenging, but two recent case reports complicated this further in CNC: in addition to the typical adrenal Cushing’s syndrome, pituitary Cushing’s disease have been also described [53, 54]. Recently aggressive corticotroph pituitary tumours and carcinomas harbouring somatic mutations in *ATRX* [55] have been described (Table 2) (Fig. 4).

### Non-functioning pituitary adenoma

**Fig. 5:**
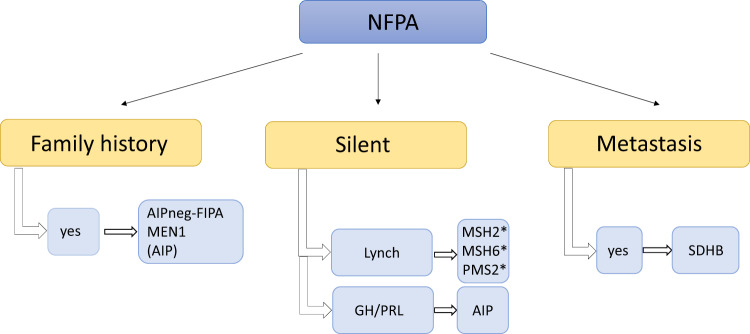
Diseases to be considered in cases of non-functioning pituitary adenoma (NFPA). AIP aryl hydrocarbon receptor-interacting protein, AIPneg-FIPA AIP mutation-negative Familial Isolated Pituitary Adenoma (this group does not represent XLAG families), FIPA Familial Isolated Pituitary Adenoma, GH growth hormone, MEN multiple endocrine neoplasia, PRL prolactin, SDH succinate dehydrogenase. *Case reports/further study needed [45, 74]

NFPAs can occur in MEN1 and mostly *AIP* mutation-negative FIPA patients. NFPA can be part of *AIP* mutation-negative homogenous NFPA kindreds—12% in our cohort of 318 FIPA kindreds, or part of heterogenous families—31% in our *AIP* mutation-negative cohort. *AIP* mutation-positive clinically NFPA cases are often microadenomas identified in asymptomatic carriers or if operated show positive GH and PRL immunostaining [56]. NFPA might be part of the 3Pa as well, and interestingly, the pituitary carcinoma case with an *SDHB* mutation was a non-functioning pituitary tumour [30]. NFPAs are most often clinically silent tumours, most often of gonadotroph origin (Fig. 5).

### Thyrotrophinoma

**Fig. 6:**
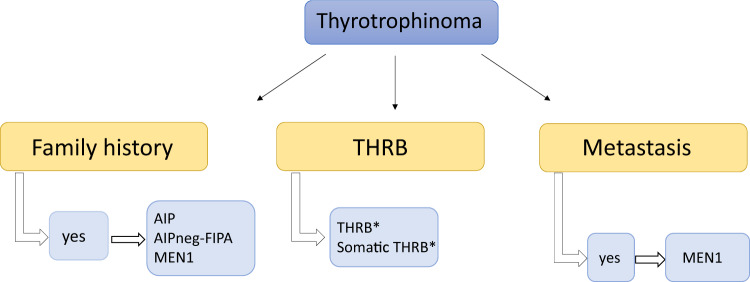
Diseases to be considered in cases of thyrotrophinomas. AIP aryl hydrocarbon receptor-interacting protein, AIPneg-FIPA AIP mutation-negative Familial Isolated Pituitary Adenoma (this group does not represent XLAG families), FIPA Familial Isolated Pituitary Adenoma, MEN multiple endocrine neoplasia, THRB thyroid hormone receptor beta. *Case reports/further study needed [62, 63]

TSH-producing pituitary tumours are very rare in a familial setting. There is one case described with an *AIP* mutation and a few cases with *MEN1* mutation [57] including an *MEN1* mutation-positive metastatic case [58]. In MEN1 syndrome PIT1-positive plurihormonal adenomas can also be identified, sometimes with silent or clinically relevant TSH positivity [59–61]. Somatic mutation has been described in TRβ (*THRB*) [62] and a patient with TSHoma and germline *THRB* mutation [63], corresponding to data from a *THRB*-deficiency animal model where TSH-secreting adenomas have been observed [64] (Fig. 6).

### Clinical considerations

Pituitary tumours in a familial setting are relatively rare disorders, and identification of the genetic cause can lead to several advantages. Discovery of a syndromic disease might help to identify other aspects of the proband’s condition with beneficial effects through earlier treatment. Genetic screening might help to identify family members in an earlier stage of the disease, when the tumour responds better to therapy and a better outcome can predicted.

First of all, a detailed family history should be obtained from all patients with a pituitary tumour. In case of FIPA, screening for *AIP* mutations should be considered, as mutations are identified in about 10% of unselected families and 20% of those with familial acromegaly. Screening for *AIP* mutations should also be considered in patients, primarily with GH or prolactin-secreting adenomas, with age at onset ≤18 years and patients with macroadenomas and age at onset ≤30 years. Genetic and clinical screening show clear benefits in *AIP* mutation-positive patients; prospectively diagnosed patients have smaller, less invasive lesions controlled less frequently with multimodal treatment, compared with clinically presenting patients [65]. XLAG should also be considered in case of early-onset gigantism starting during the first 2 years of life.

In the case of MEN1, the additional main clinical features, such as hyperparathyroidism and enteropancreatic NET present in the proband or other family members, can help in the decision regarding genetic testing. However, pituitary tumours may represent the first disease manifestation, and considering the possibility of de novo mutations, screening for MEN1 should be considered in patients with childhood-onset pituitary macroadenomas (especially prolactinomas) [66]. In patients with a MEN1 phenotype but without *MEN1* mutation, the rare MEN4 should be considered and genetic testing for *CDKN1B* mutation is suggested. Case reports of patients with multiple endocrine tumours and *CDC73* or *MAX* variants have been described.

Other less frequent syndromes should be considered in the presence of associated features, such as testing for *SDHx* mutation in case of phaeochromocytoma/paraganglioma with pituitary adenoma or testing for *PRKAR1A* in case of symptoms characteristic of CNC.

For patients with pituitary diseases there are a few questions to consider:

- *Which patient needs genetic testing?*
  Genetic testing is recommended in patients (i) with a family history of pituitary tumour, (ii) with early-onset pituitary tumour and (iii) with additional clinical features which predispose to pituitary tumour in a syndromic setting.
- *What change will bring a genetic diagnosis in the treatment or the follow-up of the proband?*
  Identifying a mutation helps to understand the nature of the disease and be more vigilant regarding tumour behaviour, can draw attention to possible hyperplasia, but in general would not necessarily change the treatment algorithm of the proband.
- *Which family members need genetic screening?*
  Genetic testing should be offered to first-degree relatives (children, siblings and parents) of a gene carrier.
- *How should carrier family members be followed up?*
  It depends on the specific syndromic condition, but in general baseline clinical, biochemical and imaging assessment is needed with follow-up depending on the specific syndrome and patient age.
  In the case of *AIP* mutation carriers, it is advised to monitor growth starting in early childhood and yearly biochemical testing (GH, IGF-1 and prolactin) not later than at the age of 10 years. A baseline pituitary MRI is advised around the age of 10 and should be repeated every 5 years until the age of 30, although between 20 and 30 follow-up could be gradually relaxed to facilitate patient cooperation. In carriers identified as adults, if a baseline clinical and biochemical assessments with pituitary MRI has not identified any abnormality, then screening can be reduced after the age of 30, as most patients with AIP mutation start their disease before this age.
  In case of MEN1 patients, published guidelines should be followed. For pituitary disease, starting at the age of 5 with yearly clinical and biochemical assessments (prolactin and IGF-1). A pituitary MRI should be performed every 3 years [67, 68].
- *If the categorisation of an identified variant is unclear, i.e., a variant of uncertain significance, what policy should be followed for the proband and family members?*

In this case, genetic testing of family members is not recommended. However, attention should be focused on any family member developing typical signs or symptoms; that person should be clinically evaluated, and if affected, genetic testing performed. As data on genetic variants are constantly evolving, patients and their clinicians should be later notified if the status of the variant has changed based on novel clinical, experimental or genetic data.

In conclusion, genetic testing not only helps us to identify patients with an increased risk of developing pituitary or other tumours, and to achieve early diagnosis and better therapeutic outcome, but also leads to better understanding of pituitary tumorigenesis.
